# Application possibilities of artificial intelligence in facial vascularized composite allotransplantation—a narrative review

**DOI:** 10.3389/fsurg.2023.1266399

**Published:** 2023-10-30

**Authors:** Leonard Knoedler, Samuel Knoedler, Omar Allam, Katya Remy, Maximilian Miragall, Ali-Farid Safi, Michael Alfertshofer, Bohdan Pomahac, Martin Kauke-Navarro

**Affiliations:** ^1^ Department of Plastic, Hand- and Reconstructive Surgery, University Hospital Regensburg, Regensburg, Germany; ^2^ Division of Plastic Surgery, Department of Surgery, Yale New Haven Hospital, Yale School of Medicine, New Haven, CT, United States; ^3^ Department of Oral and Maxillofacial Surgery, University Hospital Regensburg, Regensburg, Germany; ^4^ Craniologicum, Center for Cranio-Maxillo-Facial Surgery, Bern, Switzerland; ^5^ Faculty of Medicine, University of Bern, Bern, Switzerland; ^6^ Division of Hand, Plastic and Aesthetic Surgery, Ludwig-Maximilians University Munich, Munich, Germany

**Keywords:** vascularized composite allotransplantation, VCA, facial VCA, face transplant, artificial intelligence, AI, machine learning, deep learning

## Abstract

Facial vascularized composite allotransplantation (FVCA) is an emerging field of reconstructive surgery that represents a dogmatic shift in the surgical treatment of patients with severe facial disfigurements. While conventional reconstructive strategies were previously considered the goldstandard for patients with devastating facial trauma, FVCA has demonstrated promising short- and long-term outcomes. Yet, there remain several obstacles that complicate the integration of FVCA procedures into the standard workflow for facial trauma patients. Artificial intelligence (AI) has been shown to provide targeted and resource-effective solutions for persisting clinical challenges in various specialties. However, there is a paucity of studies elucidating the combination of FVCA and AI to overcome such hurdles. Here, we delineate the application possibilities of AI in the field of FVCA and discuss the use of AI technology for FVCA outcome simulation, diagnosis and prediction of rejection episodes, and malignancy screening. This line of research may serve as a fundament for future studies linking these two revolutionary biotechnologies.

## Introduction

Facial vascularized composite allotransplantation (FVCA) is a surgical procedure that involves transplanting composite tissue to patients with devastating and irreversible (mid-)facial defects (1–4). FVCA transplants are comprised of different tissue types such as skin, muscle, bone, nerves, and blood vessels (5). This approach represents a novel surgical therapy for complex clinical conditions (e.g., extensive burn or gunshot wounds) that cannot be effectively addressed through conventional reconstructive techniques (6). As of April 2023, there have been at least 47 FVCA worldwide with promising short- and longer-term outcomes (4, 7). Yet, FVCA research still needs to overcome different hurdles to further enhance postoperative results and increase the feasibility and accessibility of FVCA procedures. Such hurdles range from individualized preoperative planning to life-long immunosuppressive drug regimens that can increase the risk of malignancies (1, 8).

**Figure 1:**
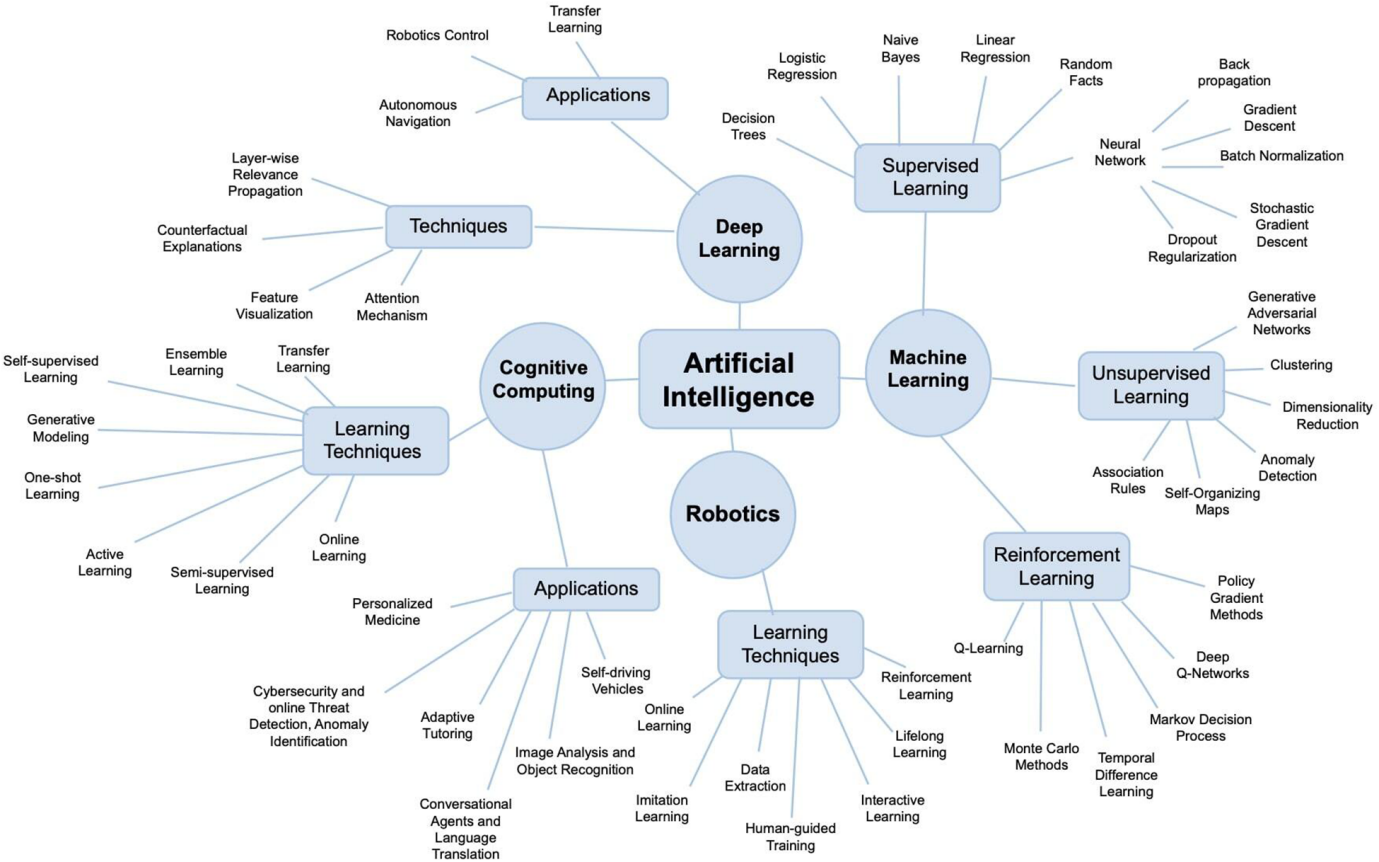
Artificial intelligence (AI) is an umbrella term for various computational strategies including machine learning, robotics, or cognitive computing. Note that, there is no scientific consensus on how to subcategorize AI and classifications may vary between different authors.

While various basic science and translational projects are underway to solve these issues, there is a scarcity of studies elucidating the integration of artificial intelligence (AI) into the field of FVCA. AI refers to computer systems that can learn, infer, synthesize, and perceive information similar to human cognition. AI is commonly established on large data sets that serve as training models to produce mathematical and computational programs; these computational designs are often based on machine learning (ML) and its subsets including deep learning (DL), and neural networks, among other computational methods (Figure 1). More importantly, AI has recently seen a rapid expansion in capabilities as computational power has exponentially increased in the past decade; however, these advancements have yet to translate into the field of FVCA.

Herein, we aim to shed light on the application possibilities of AI systems in FVCA and discuss future research directions. This scoping review may provide a fundament to leverage the innovation of FVCA and the limitless learning capacities of AI.

## AI-Aided outcome simulation

Over the past decades, preoperative surgical outcome simulation has become a vital component of comprehensive perioperative patient management (9). Preoperative outcome simulation encompasses visual and statistical prognoses of surgical procedures. Currently, the combination of patient photo series, mathematical calculation models, in-person consultation, and the surgeon's clinical experience still represent the goldstandard to provide patients with realistic outcome predictions (10, 11). Yet, AI-aided simulations have been demonstrated to automatically produce precise prognoses for various procedures in plastic and reconstructive surgery (9, 12–14).

A 2020 study by Bashiri-Bawil et al. trained an AI-based computer model to visualize postoperative rhinoplasty results using 400 patient images (frontal and side view) and 87 facial landmarks. In a testing set of 20 patients, this approach resulted in an accuracy level of >80% (15). Similarly, Bottino et al. utilized 125 face profile images to train an AI algorithm on rhinoplasties. Besides possible outcome visualization, this computer model also suggested a best-matching face profile based on the patient's individual facial characteristics. This automated, yet personalized approach yielded positive qualitative results with a quantitative assessment of postoperative attractiveness improvement still pending (16). A recent study by Mussi et al. introduced an AI-aided strategy for the preoperative simulation of autologous ear reconstruction procedures. The authors proposed a semi-automated workflowvthat integrated AI-based strategies to provide advanced design and customization techniques for this type of surgery. The authors further underscored that such easy-to-use tools may allow for streamlining the production process and reducing overall treatment costs (17). Persing et al. used the 3D VECTRA [Canfield, Fairfield, NJ; a 3D photosystem based on visual assessment management software (VAM)] to compare AI-generated to actual rhinoplasty outcomes. To this end, the authors enrolled 40 patients and concluded that this simulation technique is a powerful planning tool for rhinoplasty candidates (18).

However, such concepts cannot be directly transferred to FVCA, since preoperative outcome simulation needs to integrate the donor's and patient's facial features at the same time. Subsequently, reliable predictions can only be made once the donor is identified. Automated facial landmark recognition systems such as Emotrics (a ML algorithm that automatically localizes 68 facial landmarks) or 3D VECTRA could be used for quick and precise facial measurements (19, 20). Following facial feature recognition (e.g., nasal dorsal humps, prominent cheekbones) and exact skin color assessment, DL models [e.g., convolutional neural networks (CCN)] or VAM-based systems could generate postoperative FVCA outcome simulation and support optimal skin color matching. Of note, novel AI-based models can predict surgical outcomes based on limited datasets (i.e., less than ten observations per predictor variable) reducing the time from donor identification to outcome simulation (21). AI-aided outcome prediction may play a vital role in managing patients' expectations given that the number of FVCA performed worldwide is still limited with large interpatient variabilities. For example, there has been only one case of a Black FVCA patient (4). Previous outcome images, therefore, provide little decision support for the recipient's preoperative informed consent. Yet, future studies are warranted to establish an efficient workflow starting with donor identification over facial feature recognition to outcome simulation.

## AI-Aided intraoperative image-guidance

The potential advantages of AI are substantial, particularly for intricate procedures such as FVCA. The degree to which preoperative planning can be detailed and meticulously executed largely predicts the potential success of the outcome.

Precision and real-time feedback are essential pillars of contemporary surgical practice. These elements are considerably amplified with the integration of AI into intraoperative image-guidance systems (22). These advanced systems have the capability to discern minute details that may escape the human eye or rare anatomical variations (23). For instance, den Boer et al. proposed a DL algorithm to identify anatomical structures (e.g., aorta, right lung) based on 83 video sequences. They calculated accuracy levels of up to 90% underscoring the potential clinical applicability (24). Hashimoto et al. programmed an artificial neural network to automatically detect the cystic duct and artery yielding an accuracy of 95% (25). Such DL algorithms for landmark identification may help preserve critical anatomical structures such as the facial nerve, ultimately improving transplantation outcomes.

During FVCA, the precise fitting of the donor tissue onto the recipient's facial structure is paramount for the success of the procedure (26, 27). AI software can analyze intraoperative images in real-time, providing surgeons with detailed instructions and assisting in for example the precise positioning (22, 23). Moreover, the precision offered by AI extends beyond simple visual analysis. AI systems are capable of predicting potential risks and outcomes with increased accuracy based on patient history and health records. For instance, Formeister et al. enrolled 364 patients undergoing head and neck free tissue transfer to predict surgical complications. To this end, they trained a supervised ML algorithm to prognosticate surgical complications using 14 clinicopathologic patient characteristics (e.g., age, smoking pack-years). The ML algorithm yielded a prediction accuracy of 75% (28). Further, a meta-analysis including 13 studies concluded that CNN are sensitive and specific tools to capture intraoperative adverse events (e.g., bleeding, perfusion deficiencies) (29). These features aid surgeons in making precise and informed decisions during critical phases of the operation.

Real-time feedback plays a significant role during surgery, a role that might be efficiently fulfilled by AI-integrated intraoperative imaging systems. These systems provide surgeons with real-time data, enabling immediate adjustments to their surgical approach, which can prove invaluable during complex procedures like FVCA (30). More precisely, Nespolo et al. enrolled ten phacoemulsification cataract surgery patients and trained a deep neural network (DNN) based on the intraoperative video sequences. The DNN showed an area under the curve of up to 0.99 for identifying different surgical procedure steps such as capsulorhexis and phacoemulsification with a processing speed of 97 images per second (31). Studier-Fischer et al. trained a DNN on 9,059 hyperspectral images from 46 pigs to test its intraoperative tissue detection accuracy. The authors calculated accuracy levels of 95% when automatically differentiating 20 organ and tissue types (32). In another study of nine thyroidectomy patients, Maktabi et al. programmed a supervised ML classification algorithm to automatically discriminate the parathyroid, the thyroid, and the recurrent laryngeal nerve from surrounding tissue and surgical instruments. The mean accuracy was 68% ± 23% with an image processing time of 1.4 s (33). However, such intraoperative approaches should still be considered add-ons to the surgeon's real-time feedback rather than making hands-on experience obsolete.

## AI-Aided diagnosis and prediction of rejection episodes

Despite distinct advancements in the perioperative patient management including more patient-specific risk profiles, novel biomarkers, and targeted immunosuppressants, allograft rejection persists as a major burden in transplant surgery still affecting more than 15% of solid organ transplant (SOT) patients (34, 35). More precisely, acute rejection reactions occur in approximately 30% of liver, 40% of heart, and 33% of renal transplant recipients resulting in increased mortality rates, delayed graft function, and poor long-term outcomes (36–38). While tissue histopathology assessment represents the goldstandard for graft rejection diagnosis, it poses the downsides of low reproducibility and high inter-observer variability and has been shown to be inefficient for assessing graft steatosis, (i.e., a clinical parameter for predicting post-transplant graft function) (39, 40).

In SOT, ML and DNN (especially CNN) have been successfully investigated to identify risk patients for transplant rejection. Thongprayoon et al. performed ML consensus cluster analysis for 22,687 Black kidney transplant recipients identifying four distinct clusters. Interestingly, the authors found that the risk for transplant rejection was significantly increased in highly sensitized recipients of deceased donor kidney retransplants and young recipients with hypertension (41). In liver transplantation, Zare et al. programmed a feed-forward neural network (i.e., a type of artificial neural network where information flows in one direction from input to output) based on laboratory values and clinical data from 148 recipients. The network outperformed conventional logistic regression models in predicting the risk of acute rejection seven days after transplant, with 87% sensitivity, 90% specificity, and 90% accuracy and correctly determined eight out of ten acute rejection patients and 34 out of 36 non-rejection ones in the testing set (42). The combination of different AI methods (i.e., ML and DNN) and microarray analysis also have been deployed to detect molecular signatures correlating with rejection patterns in lung transplant transbronchial biopsies and heart transplant recipients. Given the patterns' complexity, such prediction pathways only have become available since the advent of AI and the ability to make sense of enormous data set (43–45). Further, CNN have been demonstrated to facilitate the diagnostic workflow for transplant rejection. Abdeltawab et al. merged data from 56 patients (e.g., diffusion-weighted MRI images and clinical biomarkers such as creatinine clearance and serum plasma creatinine) to predict kidney graft rejection with an accuracy of 93%, sensitivity of 93%, and specificity of 92% (46).

Across the different vascularized composite allograft (VCA) surgery subtypes, 85% of VCA patients experience at least one rejection episode one year post transplantation with more than 50% of cases reporting multiple rejection episodes (47). Such episodes can lead to irreversible loss of graft function and represent a major hurdle for integrating FVCA surgery into routine clinical care (48). Current diagnosis protocols recommend a prompt biopsy and histologic examination in case of suspicious cutaneous changes. For histologically confirmed rejection reactions, immediate steroid bolus administration and/or other immunosuppressants are commonly administered (49). Besides a more standardized method for prediction and diagnosis of FVCA rejection, AI models could allow for non-invasive graft examination based on distinct macroscopic changes typical of transplant rejection (e.g., a maculopapular erythematous rash of varying color intensities) (50–52). For instance, a CNN classifier could be trained based on FVCA patient images to detect rejection episodes. Skin photographs of patients with transplant rejection compared to healthy individuals may serve as input data (48, 53, 54). Alternatively, patient mucosa photographs could be utilized for training purposes since the mucosal tissue was recently identified as one of the primary target sites for rejection episodes by Kauke-Navarro et al. (2, 55). Given the limited number of FVCA procedures, researchers may integrate data augmentation techniques such as flipping (i.e., horizontally or vertically mirroring an image or data point), shearing (i.e., distorting the image by tilting it in a specific direction), or color channel shifting (i.e., altering the intensity of individual color channels in the image) (56, 57). Further, computer vision (CV) could be used to extract visual data from patient images or videos. Following image classification, detection, and recognition, CV may autonomously analyze and interpret signs of VCA rejection (58, 59). Moreover, AI technology (e.g., CNN) may support large data storage and maintenance through automatized deduplication and error detection, ultimately leading to a more holistic approach to rejection prediction and diagnosis. Such a database could integrate genetic, metabolomic, immunoproteomic, histological variables, and laboratory values to leverage their diagnostic and predictive value (60, 61).

## AI-Aided malignancy screening

Cancer represents a widely recognized complication of both SOT and VCA, since there is a two to four-fold elevated risk of malignancies following organ transplant. Remarkably, transplant recipients are at a 100-fold higher risk for developing skin cancer (62). While the exact mechanisms are still under investigation, immunosuppressants have been linked to an increased incidence of cancer in transplant recipients when compared to age-matched control groups (63). Of note, different regimes of immunosuppressants resulted in comparable cancer rates (64). Therefore, follow-up guidelines commonly include routine check-ups such as skin examinations (65). Yet, there are various barriers for access to such dermatologic care (e.g., long wait times, lack of specialized transplant dermatologists, long travel distances) and aftercare in general that reduce patient compliance and the effectiveness of follow-up care (66).

AI technology (especially CNN and DL) has been shown to provide a versatile and precise screening platform for diverse cancer entities and detect mammographic abnormalities with comparable accuracy to radiologists and reduce radiologist workloads (67–70). Yi et al. programmed DeepCAT (a DL platform) including two key elements for mammogram suspicion scoring: (1) discrete masses and (2) other image features indicative of cancer, such as architectural distortion. The authors used a training set of 1,878 2D mammographic scans. The model classified 315 out of 595 images (53%) as “low priority”. Notably, none of these low-priority images contained any tumorous malignancies (71). Moreover, Kulkarni et al. used digital pathology images of 108 breast cancer patients to predict disease-specific survival. The authors reported an area under the curve of 0.90 in their testing set which consisted of 104 patients. Based on this cut-off, the CNN model could reliably assess the disease-specific survival (72). Lotter et al. developed a DL-aided diagnostic tool to effectively screen mammographies. Strikingly, the algorithm outperformed five radiologists, each fellowship-trained in breast imaging, on a cancer-enriched dataset. The algorithm resulted in an absolute increase in sensitivity of 14% and specificity of 24%, respectively. Further, the AI-based approach showed promising potential to detect interval cancers (increase in sensitivity of 18%; increase in specificity of 16%) (73). Tschandl et al. further used ML to categorize skin neoplasia. When comparing the screening accuracy of 511 human evaluators (dermatologists, general practitioners) to the AI model, the authors found that the algorithm yielded a significantly higher accuracy. Interestingly, the AI platform even outperformed dermatologists with more than ten years of clinical experience (74). For skin cancer screening, AI yielded an average accuracy value of 87% (with a maximum of 99% and a minimum of 67%) (75–79). Further, Pham et al. programmed a ML platform, based on 17,302 images of melanoma and nevus. The model performance was compared to that of 157 dermatologists from twelve university hospitals in Germany. The authors reported that the AI-based screening approach yielded a sensitivity of 85%, and specificity of 95%, ultimately outperforming all human evaluators (80). DL has also been demonstrated to reliably detect soft tissue sarcomas using 506 histopathological slides from 291 patients. The screening program showed an accuracy of 80% in diagnosing the five most common soft tissue sarcoma subtypes, as well as significantly improving the accuracy of the pathologists from 46% to 87% (81).

**Figure 2:**
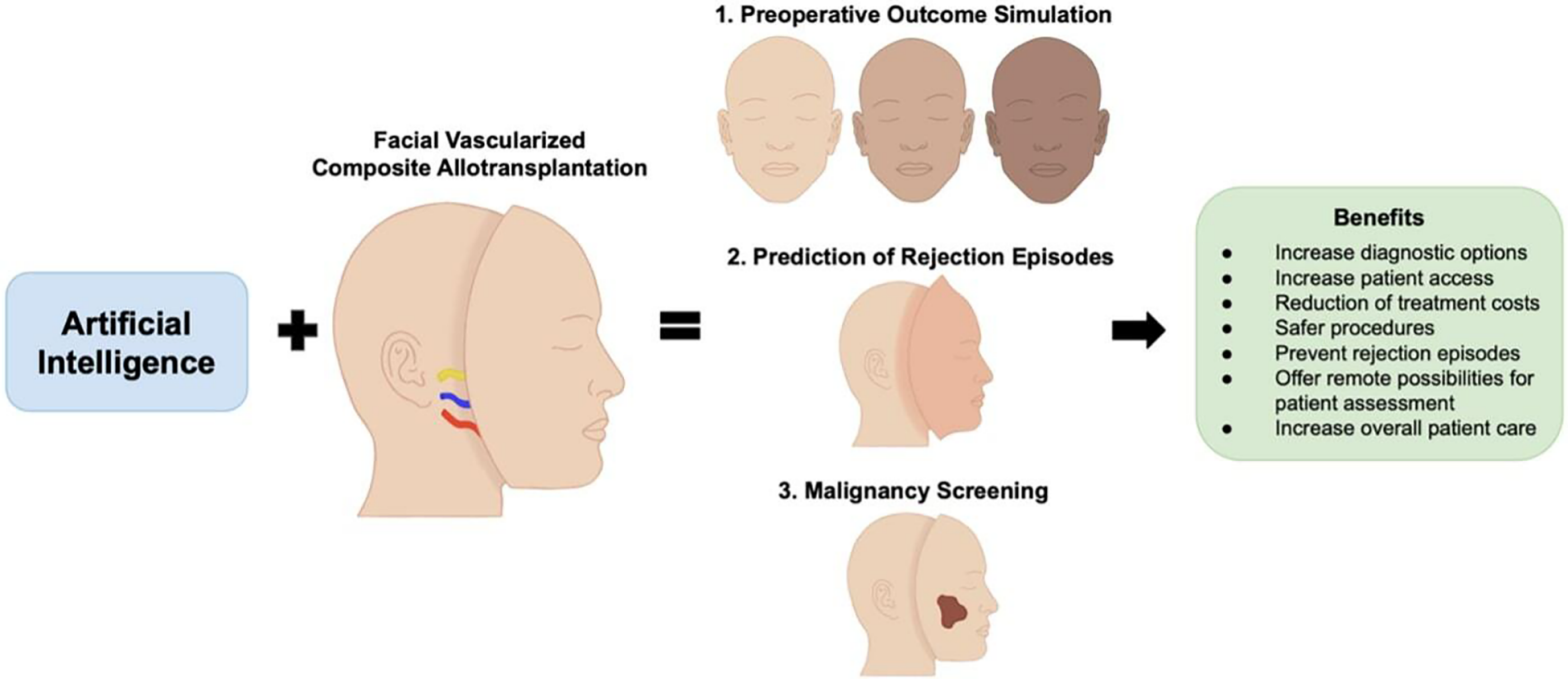
The combination of artificial intelligence and facial vascularized composite allotransplantation carries promising potential in advancing preoperative outcome simulation, prediction and diagnosis of rejection episodes, and cancer screening.

Following FVCA, one transplant recipient patient was diagnosed with basal cell carcinoma of the native facial skin six years post-transplant, while another FVCA patient presented with a B cell lymphoma which was treated with rituximab, cyclophosphamide, vincristine, doxorubicin, and prednisolone (82). Despite the successful treatment of the lymphoma, the patient was diagnosed with posttransplant-related liver tumors (83). Vice versa, there were also two cases of patients who underwent FVCA because of their cancer disease. The first was a Chinese patient with advanced melanoma who received a graft including the scalp and both ears. Of note, the long-term outcome of this case remains unreported (84). The other case involved a 42-year-old, HIV-positive male from Spain. While pseudosarcomatous spindle cell proliferation was diagnosed eleven months postoperatively and successfully treated, the patient ultimately died of a transplant-related lymphoma (85). AI technology carries promising potential to address this medical complication by facilitating posttransplant screening examinations and allowing for more frequent follow-up checks. Given that FVCA still remains a novel procedure with a specific indication, AI-aided cancer screening could also accelerate the examiner's diagnosis-making, thus leveraging AI and the limited human clinical experience. This semi-automated, human-controlled pathway may represent a valuable alternative to fully automatized strategies when discussing possible liability issues (Figure 2) (86).

## Discussion

FVCA has emerged as a novel and versatile reconstructive technique to provide adequate therapy for patients with devastating facial wounds and trauma (61, 87). While the surgical outcomes have proven its effectiveness, further steps are needed to streamline the perioperative workflow in FVCA surgery, thus broadening access to this type of surgery and increasing the work volume (7). Further research is needed to overcome persisting hurdles in the field. For instance, AI performance is based on access to patient datasets used for training. However, recent research has underpinned that AI algorithms could be susceptible to data security breaches (88). Given that FVCA involves highly sensitive patient information that allows to identify FVCA patients, any data breach could have severe ethical and legal implications. Therefore, the integration of AI into the field of FVCA calls for comprehensive data security strategies at the same time. Moreover, Obermeyer et al. revealed racial disparities in a state-of-the-art U.S. healthcare algorithm. The authors found that Black patients were in significantly poorer condition compared to White patients assigned the same level of risk by the algorithm, ultimately reducing the number of Black patients receiving extra care by ∼50% (89). Our group has recently highlighted the risk of racial disparities in FVCA patients (90). Therefore, FVCA providers should be sensitized on AI technology potentially catalyzing racial inequities. To this date, FVCA surgery still represents a highly individualized and resource-intensive therapy concept. Therefore, AI could help reduce work costs and time, as well as address persisting challenges in this uprising field such as outcome simulation, rejection detection, and postoperative cancer screening. This line of research may serve as the fundament for large-scale studies investigating possible points of leverage between FVCA and different AI concepts. Future research areas may include the use of AI in personalized drug therapy following FVCA. AI algorithms such as neural networks are being used to tailor drug regimens (i.e., drug combinations, drug dosages) in liver transplantation patients (NCT03527238). This approach could reduce the therapy-induced side effects after FVCA and expand the FVCA recipient pool onto patients with severe preexisting comorbidities resulting in complex drug interaction (91). In addition, AI-driven natural language processing systems could facilitate remote monitoring of FVCA patients. There is evidence that AI-supported telemedicine helps improve patient-reported outcomes in chronic disease care (e.g., diabetes mellitus type II, malignancies) (92, 93). Currently, at least 23 U.S. institutions have established FVCA programs with some patients traveling long-distance for pre- and postoperative clinic visits (94). Long distances between healthcare providers and patients have been demonstrated to reduce compliance leading to poorer postoperative outcomes (95). AI-powered telemedicine may fill in this healthcare gap facilitating closer perioperative FVCA patient management. Overall, the symbiosis of FVCA and AI carries untapped potential for improving patient healthcare and advancing this revolutionary surgical approach.
